# Haptoglobin and Hemopexin Redirect Heme-Driven Oxidative Stress and Neurotoxicity in Organotypic Brain Slices

**DOI:** 10.1021/acschemneuro.5c00511

**Published:** 2025-12-19

**Authors:** Anna-Lea T. Stalder, Raphael M. Buzzi, Florence Vallelian, Dominik J. Schaer

**Affiliations:** Department of Internal Medicine, Universitätsspital and University of Zurich, Zurich 8091,Switzerland

**Keywords:** hemorrhagic stroke, hemoglobin, heme, haptoglobin, hemopexin, organotypic brain slice culture, secondary brain injury

## Abstract

Hemorrhagic stroke triggers secondary brain injury through the red blood cell toxins hemoglobin (Hb) and heme, which fuel iron-driven lipid peroxidation and neuronal injury. We sought to use organotypic brain-slice cultures to dissect how the high-affinity scavenger proteins haptoglobin (Hp) and hemopexin (Hpx) modulate this cascade. By day 7 of culture, slices remained structurally intact, metabolically active, and responsive to oxidative stress, enabling precise toxin exposure studies. Isotopic ^58^Fe tracing revealed that upon cell-free Hb and heme exposures, heme-iron accumulated in brain slices and heightened lipid peroxidation. In contrast, Hpx neutralized heme, nearly abolishing iron deposition, while Hp partially reduced Hb-driven iron accumulation. Both scavengers attenuated lipid peroxidation and reduced neuronal cell death. Transcriptomic profiling revealed that free toxins increased oxidative stress and neuroinflammatory activation markers, whereas Hpx suppressed the expression of heme-induced genes. Remarkably, HbHp complexes triggered a strong Nrf2-centered adaptive program that enhanced iron metabolism and glutathione synthesis. Integrating five readouts—iron accumulation, lipid peroxidation, neuronal cell death, heme-stress transcripts, and Nrf2/metabolic transcripts—via bootstrap-based principal component analysis yielded two orthogonal axes. An oxidative toxicity axis (PC1) captured iron-driven reactive oxygen species and cell death, while a metabolic adaptation axis (PC2) reflected Nrf2-mediated reprogramming. Free toxins clustered at the toxic extreme on PC1, and heme–Hpx aligned near baseline. HbHp shifted slices upward on PC2, reducing neuronal loss through safe adaptation. These findings establish that Hpx neutralizes free heme, whereas Hp stabilizes Hb and elicits cytoprotective gene expression, offering a rational, dual-scavenger strategy to mitigate secondary brain injury in hemorrhagic stroke.

## Graphical abstract

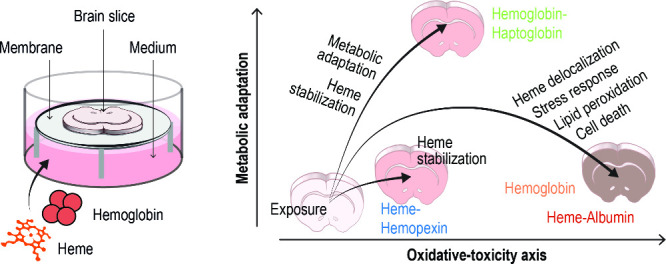

## Introduction

Intracerebral and subarachnoid hemorrhages—collectively hemorrhagic strokes—are devastating. Although they account for only ∼15–20% of all cerebrovascular events in high-income countries, they cause a disproportionate share of mortality and disability.^1,2^ Unlike ischemic strokes, which have established treatments like thrombolysis and thrombectomy, effective acute interventions for hemorrhagic strokes are limited. Despite advances in acute neurosurgical and critical-care management, more than half of all patients die within the first year, and survivors frequently sustain severe neurological sequelae that demand lifelong care.^1,3^ A major contributor to this poor prognosis is secondary brain injury (SBI), which is caused by a delayed, self-propagating cascade that unfolds once the primary bleed has been stabilized. Within this multifactorial pathophysiology, the red blood cell (RBC) products hemoglobin (Hb) and heme, while indispensable in their physiological intracellular context, contribute as potent RBC toxins when released into the extracellular space as unshielded species.^4–9^

Within the first few hours after bleeding, extravasated RBCs begin to lyse,^4,10^ releasing hemoglobin into the extracellular environment of the hematoma. Oxidative autodegradation of Hb produces catalytically active ferric species and liberates the prosthetic heme group. Free heme, with its central iron atom, readily partitions into lipid-rich cellular membranes where it fuels iron-dependent reactive oxygen species (ROS) formation, lipid peroxidation, and, ultimately, cell death.^11^ However, even though the sequence of Hb → heme → lipid ROS → cell death is now considered a key driver of neuronal loss and functional decline after hemorrhagic stroke, it has never been experimentally delineated, providing a unique field for model development and investigation.^4^

Evolution has equipped the circulation with two high-affinity scavenger proteins that intercept this destructive cascade.^11^ Haptoglobin (Hp) binds Hb in a large protein complex with femtomolar affinity,^12,13^ stabilizing the tetramer^14,15^ and facilitating receptor-mediated clearance.^15,16^ In contrast, hemopexin (Hpx) sequesters free heme in an essentially irreversible complex that prevents membrane infiltration and Fenton chemistry.^17^ However, after a hemorrhagic stroke, the natural abundance of both scavengers in the CNS is orders of magnitude below what is needed for protection, necessitating therapeutic administration to achieve beneficial levels.^18^ Preclinical rodent studies confirm that Hp or Hpx can reduce edema, oxidative stress, and neurological deficits.^5,19–21^ Yet, the cellular mechanisms that underlie this protection and the extent to which the two scavengers modulate both toxicity and adaptive stress responses remain incompletely understood.

Addressing these questions in vivo is challenging: hematoma composition, blood–brain barrier integrity, and inflammatory cell infiltration vary widely between animals, obscuring cause–and–effect relationships. Organotypic brain slice cultures (BSCs) provide an attractive ex vivo alternative, modeling the post-blood−brain barrier parenchymal environment that follows intracranial bleeding, when neurons and glia are directly exposed to cell-free Hb and heme.^22^ Thin vibratome sections preserve the cytoarchitecture, synaptic connectivity, and glia–neuron interactions of the brain, while allowing precise control over incubation conditions, toxin concentration, and exposure time. Importantly, BSCs satisfy the 3 Rs of animal welfare by replacing in vivo experimentation for mechanistic work.

In the present study, we utilized the BSC platform to reproduce the entire RBC-toxin cascade and to elucidate how Hp and Hpx influence each step of this process. We combined five complementary readouts, including heme-iron immobilization in lipid compartments, lipid peroxidation, transcriptional heme-stress signaling, adaptive heme metabolism gene expression, and neuronal cell death, and integrated them using principal-component analysis (PCA). This multidimensional approach yielded two orthogonal biological axes with an oxidative-toxicity axis dominated by iron burden and lipid peroxidation, contrasting a metabolic-adaptation axis driven by Nrf2/heme metabolism transcripts. Heme and Hb exposures clustered on the toxic extreme, whereas their respective Hpx and Hp complexes mapped to neutral and adaptive quadrants within our PCA biplot, revealing a bifurcation between injury and adaptation.

By delineating this sequential toxicity-to-adaptation landscape, we aim to provide a mechanistic rationale for targeting scavenger pathways as an adjuvant therapy for hemorrhagic stroke-associated SBI.

## Results and Discussion

### Baseline Integrity of Organotypic Brain-Slice Cultures

Organotypic brain slice cultures maintained their cytoarchitecture and metabolic competence, remaining viable for at least 14 days ex vivo (Figure 1A). By day 7, NeuN/Hoechst staining confirmed intact cortical lamination without edge necrosis (Figure 1B). Biochemical leakage mirrored these histological observations: After the initial slicing trauma, lactate-dehydrogenase (LDH) release dropped 4-fold from 11,467 ± 1471 RFU to 4,430 ± 1193 RFU between days 4 and 7 and then stabilized below 15% of the maximal signal measured after complete tissue lysis with Triton X-100 after 14 days in culture (Figure 1C). Metabolic competence was verified with a luciferin–luciferase reporter. A DMNPE-caged luciferin/luciferase reporter was employed because its light output requires intact intracellular esterases, ATP, and oxygen, providing a rapid, nondestructive readout of slice metabolic competence (Figure 1D). On day 7, live brain slices from CAG-Luc mice supplied with DMNPE-caged luciferin emitted strong bioluminescence, whereas freeze-killed controls showed only background signal. (Figure 1E,F). Importantly, continuous H_2_O_2_ generation with glucose oxidase resulted in a dose-dependent decline in bioluminescence (Figure 1G), demonstrating that the model remains responsive to redox stress. Together, these data designate day 7 as the window in which slices are structurally preserved, metabolically active, and sufficiently sensitive to detect toxin-induced oxidative injury. Therefore, all subsequent experiments were initiated on day 7 of culture.

**Figure 1:**
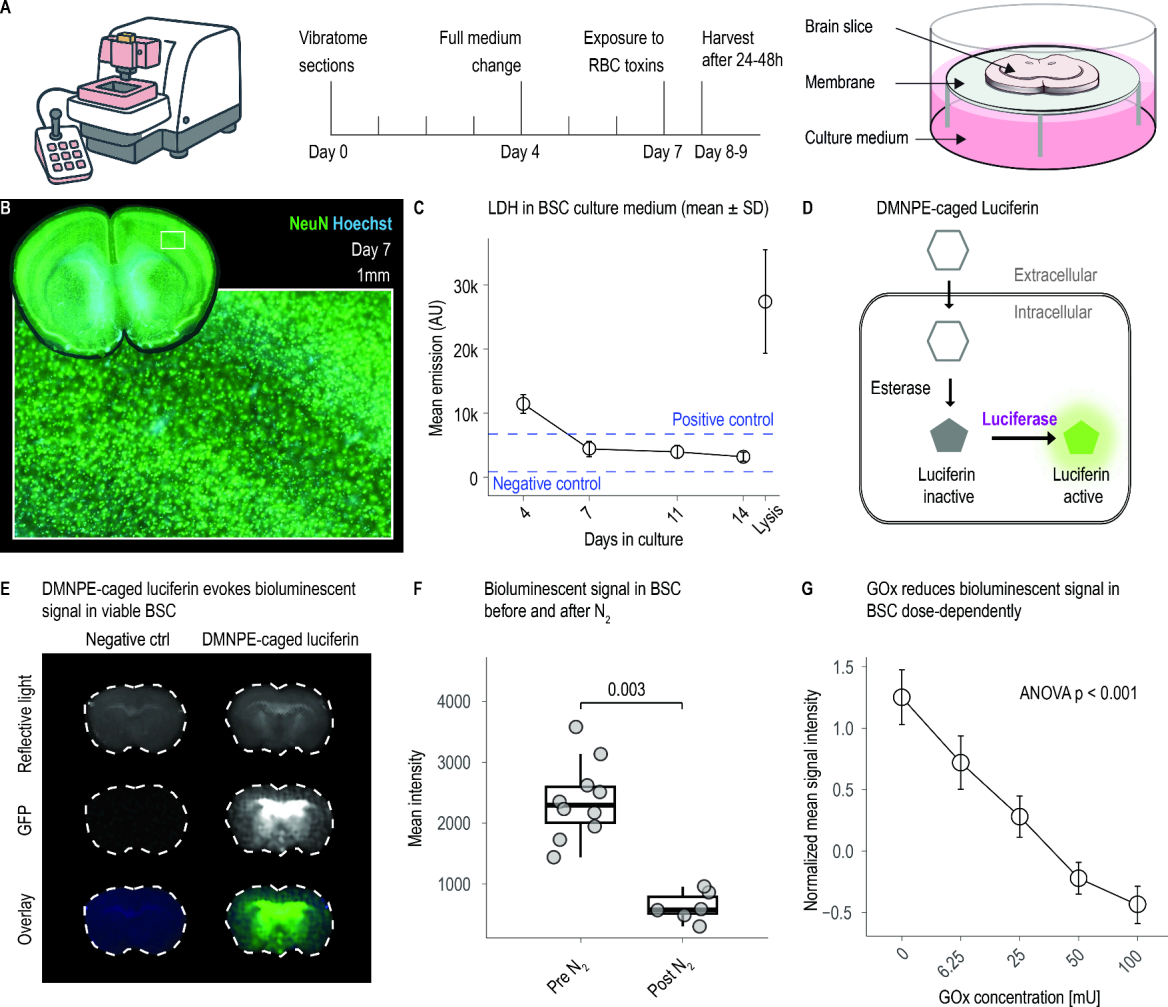
Baseline integrity and redox responsiveness of organotypic brain-slice cultures. (A) Workflow for coronal mouse brain slice preparation, air–liquid cultivation, and the experimental window (day 7). (B) NeuN (green)/Hoechst (blue) staining on culture day 7 shows preserved cortical lamination without edge necrosis. Rectangle width = 1 mm. (C) Lactate-dehydrogenase (LDH) release declines from the slicing-induced peak between days 4 and 7 and stabilizes below 15% of the Triton X‑100 lysis control. *n* = 8–24 inserts per time point; mean ± SD. (D) Principle of the DMNPE-caged luciferin assay: intracellular esterases liberate luciferin, enabling ATP- and O_2_-dependent photon emission by endogenous luciferase. (E) Representative overlays (bottom), raw luminescence (middle), and reflective light (top) images from live day-7 slices with and without DMNPE-caged luciferin. (F) Quantification of bioluminescence on day 7; *n* = 10 per group; two-tailed *t*-test, *p* = 0.003. (G) Continuous H_2_O_2_ generation with glucose oxidase (GOx, 6.25–100 mU mL^–1^) provokes a dose-dependent reduction in normalized luminescence; *n* = 12–20 slices per dose; one-way ANOVA with Tukey post hoc.

### Heme-Iron Immobilization Reveals Differential Toxin Uptake

Toxin exposure served as the entry point for a cascade of destructive assays, schematically depicted in Figure 2A. Slice sets were treated with either free Hb, heme bound to its natural low-affinity carrier protein albumin (heme-albumin),^14,23^ or their stoichiometric high-affinity scavenger complexes with Hp and Hpx, respectively. After incubation, slices were allocated to one of five readouts: ^58^Fe ICP-MS (heme-iron immobilization), TBARS/MDA (lipid peroxidation), bulk RNA-seq (transcriptional stress and adaptation scores), or immunohistochemistry for cleaved Caspase-3/NeuN (neuronal death). Because each assay consumes the tissue, separate slice batches were investigated for each assay under highly standardized conditions, allowing the five parameters to be integrated later by bootstrap-PCA.

**Figure 2:**
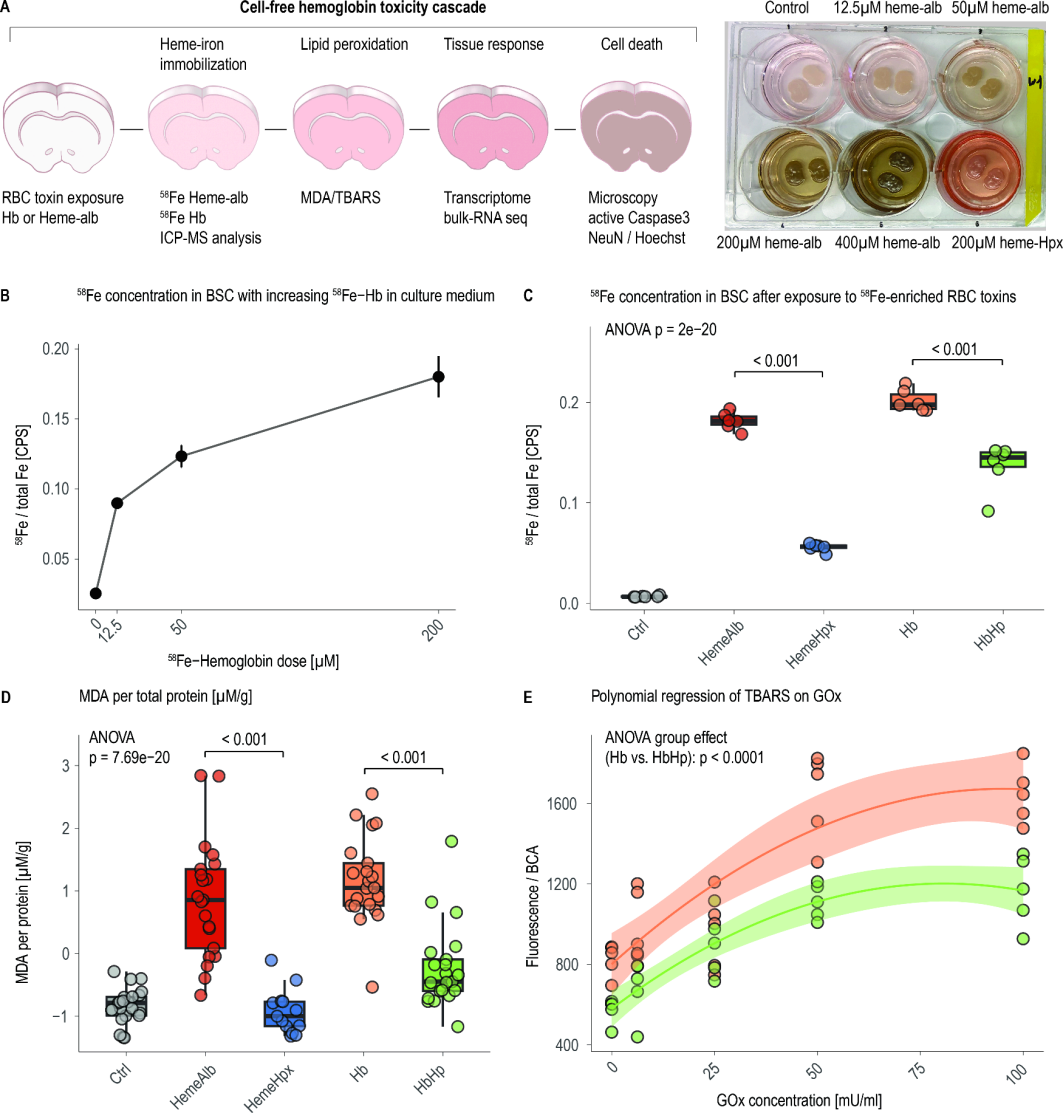
Scavenger proteins attenuate heme-iron deposition and lipid peroxidation in brain-slice cultures. (A) Experimental cascade: slices are exposed to Hb or heme-albumin with or without Hp or Hpx, followed by sequential read-outs for iron immobilization (^58^Fe ICP-MS), lipid peroxidation (TBARS/MDA), transcriptomic stress signatures, and neuronal death (cleaved Caspase-3/NeuN). The photograph shows an experimental setup with characteristic color changes of heme-albumin dilutions and hemeHpx complexes. (B) Dose–response curve for tissue ^58^Fe after 24 h exposure to 12.5–200 μM ^58^Fe-Hb; mean ± SD, *n* = 3 per dose. (C) Tissue iron burden at 200 μM toxin. Box-and-whisker plots show median, IQR, and individual points; one-way ANOVA with Tukey post-test; *n* = 6 per group. (D) TBARS fluorescence normalized to protein mirrors the iron deposition pattern; one-way ANOVA with Tukey post-test; *n* = 13–21 per group. (E) Under continuous exogenous H_2_O_2_ generated by 6.25–100 mU mL^–1^ glucose oxidase, lipid peroxidation rises but remains lower for HbHp (green); shaded areas are 95% confidence bands; overall group effect *p* < 0.0001 (polynomial regression; ANOVA).

Isotopic tracing coupled with ICP-MS quantified how much heme-iron partitions into lipid-rich tissue compartments (Figure 2A). Therefore, we used ^58^Fe-Hb and heme-albumin generated by metabolic labeling in iron-deficient mice^24^ to expose BSC and quantify tissue heme-iron deposition. Increasing doses of cell-free ^58^Fe-Hb (12.5–200 μM) produced a monotonic rise in slice iron, from 0.015 ± 0.005 ppb to 0.167 ± 0.019 ppb (*n* = 3 per dose; *p* < 0.01; Figure 2B). At an equimolar compound concentration in culture medium of 200 μM, free Hb yielded the highest iron burden (0.202 ± 0.011 ppb; *n* = 6), closely followed by heme-albumin (0.182 ± 0.009 ppb; *n* = 6). Complex formation with the cognate scavengers markedly attenuated iron uptake. Heme-Hpx restored near-baseline levels (0.056 ± 0.004 ppb; *p* < 0.001 vs heme-albumin), whereas HbHp achieved a partial but significant reduction to 0.136 ± 0.023 ppb (*p* < 0.001 vs free Hb; Figure 2C). Thus, while both scavengers blunt toxin uptake, Hpx virtually abolishes heme deposition. In contrast, Hp attenuates but does not entirely prevent Hb-derived tissue iron loading, potentially reflecting cellular uptake of HbHp complexes.

### Scavenger Proteins Blunt Heme-Driven Lipid Peroxidation

Oxidative damage to membrane lipids, measured by malondialdehyde (MDA) levels after 24 h of toxin exposure, closely mirrored the iron-immobilization pattern (Figure 2D).

Heme-albumin and free Hb increased normalized MDA from −0.83 ± 0.286 μM g^–1^ (controls, *n* = 21) to 0.861 ± 0.94 μM g^–1^ (*n* = 21) and 1.164 ± 0.685 μM g^–1^ (*n* = 21), respectively (*p* < 0.001 vs Ctrl for both). Complex formation with the scavengers sharply reduced lipid peroxidation. Heme-Hpx lowered normalized MDA to −0.924 ± 0.359 μM g^–1^ (*n* = 13, *p* < 0.001 vs heme-albumin) and HbHp to −0.234 ± 0.656 μM g^–1^ (*n* = 21, *p* < 0.001 vs Hb).

To probe the resilience of Hp protection under sustained oxidant pressure, we superimposed continuous H_2_O_2_ generation via glucose oxidase.^25^ Polynomial regression revealed a dose-dependent rise in thiobarbituric-acid-reactive substances (TBARS) fluorescence for both Hb and HbHp, yet the Hp complex remained consistently lower across the series (ANOVA group effect (Hb vs HbHp), *p* < 0.0001; Figure 2E). Hence, while free toxins drive lipid peroxidation in proportion to their iron burden, both scavenger proteins intercept this step—Hpx by neutralizing heme altogether and Hp by curtailing Hb-derived ROS even under exogenous oxidative stress.

### Neuronal Cell Death Scales with the Oxidative Load

To determine whether the biochemical signatures translate into functional injury, we quantified neuronal apoptosis. Brain slices were immunostained for NeuN and cleaved Caspase-3, followed by fully automated single-cell segmentation and k-means clustering of intensity features, providing a pseudocytometric analysis (Figure 3A,B). Two discrete populations emerged: Casp3^high^/NeuN^low^ (apoptotic) and Casp3^low^/NeuN^high^ (viable).

**Figure 3:**
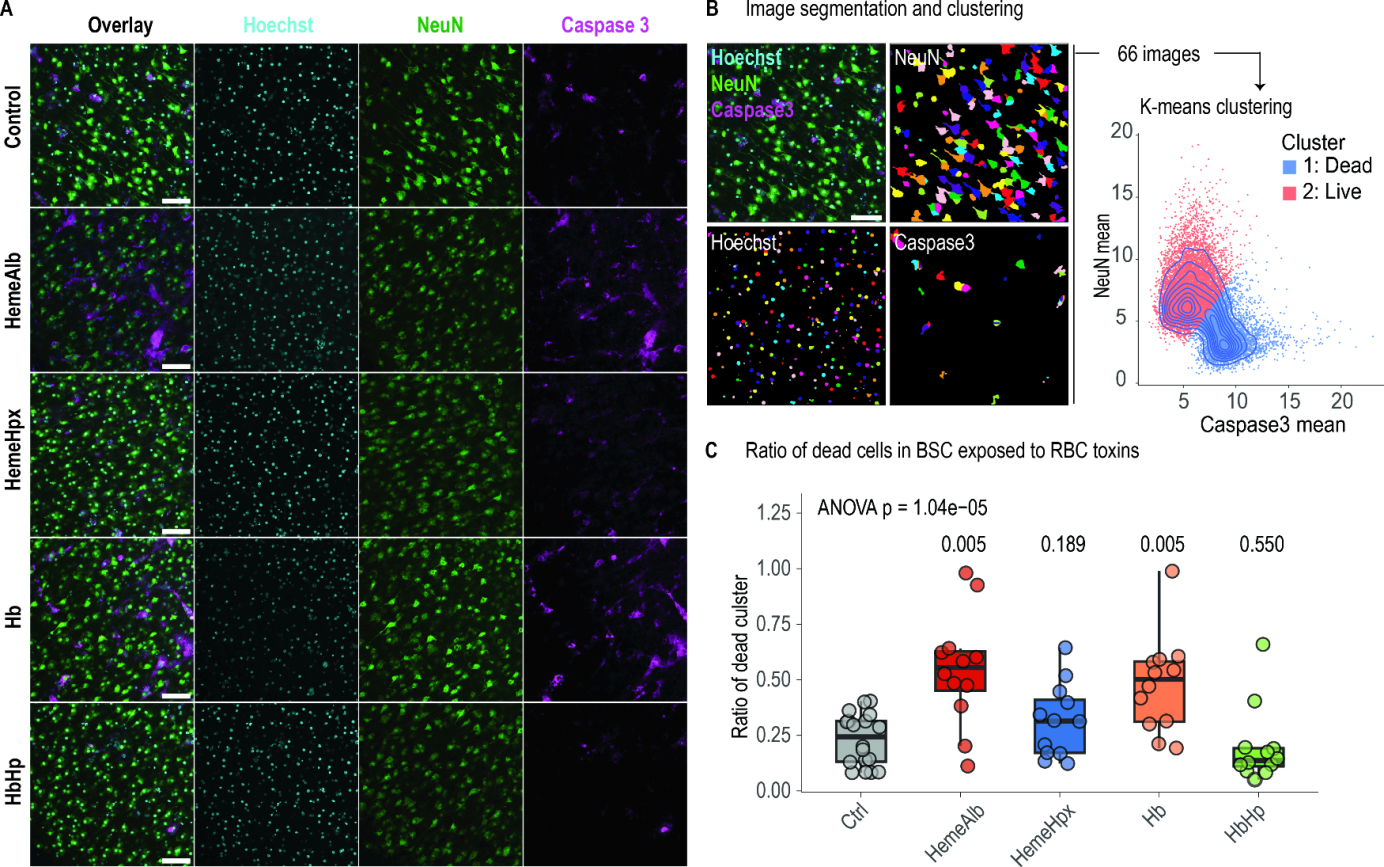
Scavenger proteins prevent toxin-induced neuronal cell death. (A) Representative confocal micrographs 24 h after exposure to RBC toxins or their scavenger complexes. Overlay and single channels are shown for Hoechst (cyan), NeuN (green), and cleaved Caspase-3 (magenta); scale bars = 50 μm. (B) Pseudocytometric quantification pipeline: single-cell segmentation of NeuN and Caspase-3 fluorescence, followed by *k*-means clustering (*k* = 2), separates Casp3^high^/NeuN^low^ dead neurons (blue) from Casp3^low^/NeuN^high^ viable neurons (red). (C) Fraction of apoptotic neurons. Box plots display median, IQR, and individual slice values (*n* = 13–18). One-way ANOVA with Tukey post-test of each treatment vs Ctrl. Free heme-albumin and Hb significantly increased apoptosis, while Hpx- and Hp-complexed toxins did not. Neuronal death, therefore, scales with oxidative load from free toxins and is mitigated when their cognate scavenger proteins sequester Hb or heme.

At baseline, 22.9 ± 12% of neurons fell into the apoptotic cluster (Figure 3C). Exposure to free toxins nearly doubled this fraction: heme-albumin increased apoptosis to 54.3 ± 25% (*p* = 0.005 vs control; *n* = 15) and free hemoglobin to 48 ± 22% (*p* = 0.005; *n* = 18). In marked contrast, scavenger complexes did not elevate cell death. Heme-Hpx maintained apoptosis at 31.4 ± 16% (n.s. vs control; *n* = 13), while HbHp reduced the index to 19.7 ± 17% (n.s. vs control; *n* = 17) and was significantly lower than free Hb (*p* < 0.01).

Representative confocal microscopy images corroborated these quantitative findings with robust Caspase-3 staining and shrunken nuclei in heme-albumin- and Hb-treated slices, whereas Hp- and Hpx-protected slices preserved neuronal morphology (Figure 3A). Thus, neuronal loss scales with iron burden and lipid peroxidation, while both scavenger proteins interrupt this cascade, with Hpx acting through toxin neutralization and Hp providing an additional adaptive component that may even repress baseline cell death.

### Transcriptomic Signatures Distinguish Oxidative Stress from Adaptive Reprogramming

Bulk RNA-seq was performed after exposure to Hb or HbHp and heme–albumin or hemeHpx, all at 200 μM. Differential expression, pathway enrichment, and the two composite gene-set scores that feed the downstream multivariate analysis are summarized in Figures 4–6.

**Figure 4:**
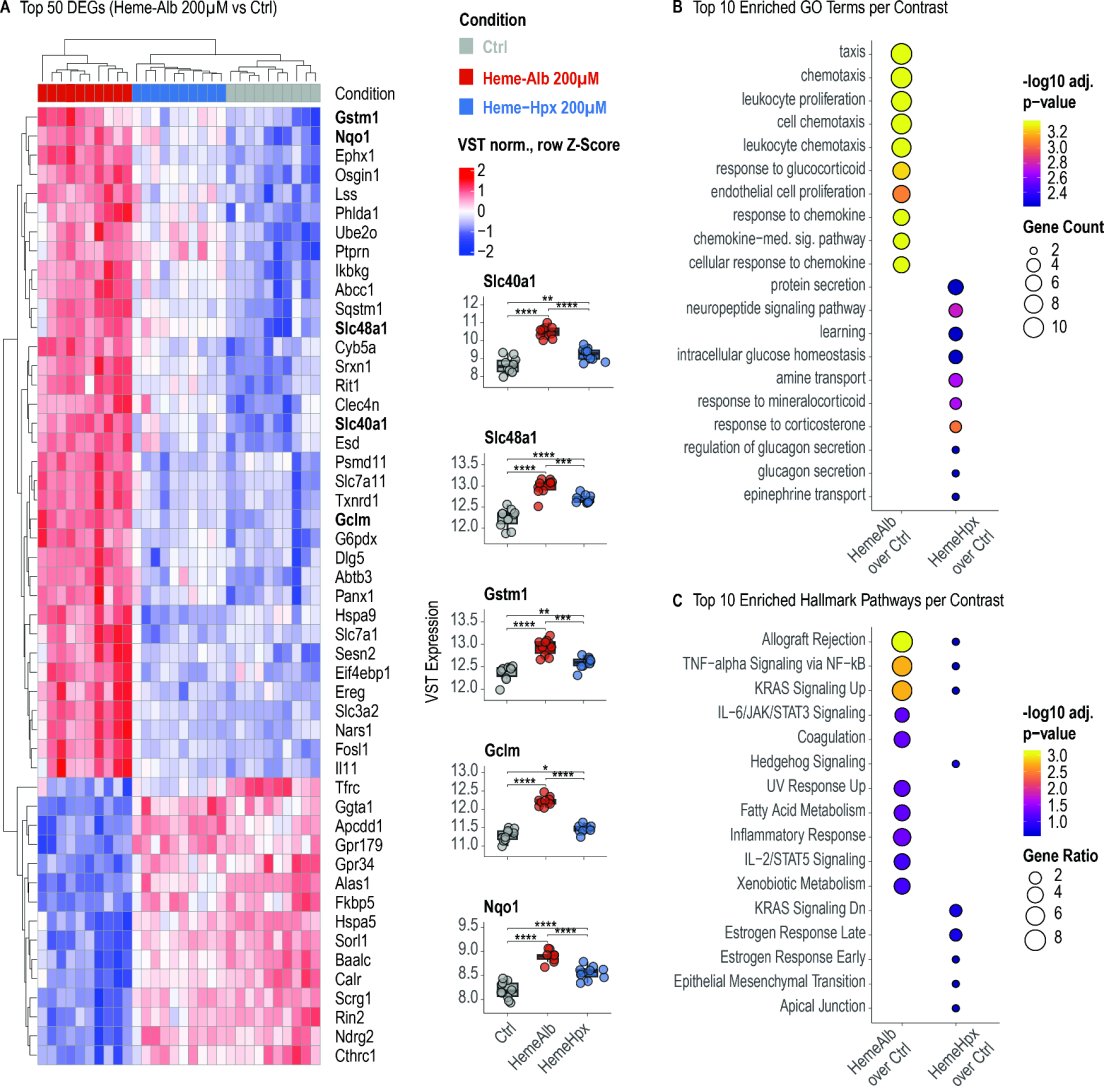
Heme–hemopexin attenuates transcriptional stress responses induced by heme–albumin. (A) Heatmap displaying top 50 DEGs following 24-h exposure to 200 μM heme–albumin, hemeHpx, or vehicle control (contrast: heme-albumin vs control). Key oxidative stress response genes (e.g., *Nqo1*, *Gclm*, *Gstm1*) and heme–iron transporters (*Slc48a1*, *Slc40a1*) were strongly upregulated by heme–albumin, whereas simultaneous hemeHpx exposure prevented this response. (B) GO enrichment analysis of DEGs following heme-albumin and hemeHpx exposure. Heme–albumin enriched oxidative stress and neuroinflammatory pathways, while Hpx shifted enrichment toward neuroendocrine and metabolic GO terms. (C) Enrichment of Hallmark gene sets. Lipid peroxidation-related and redox stress pathways were enriched in response to heme–albumin, whereas Hpx mitigated this response and promoted transcriptional profiles associated with cellular adaptation.

**Figure 5:**
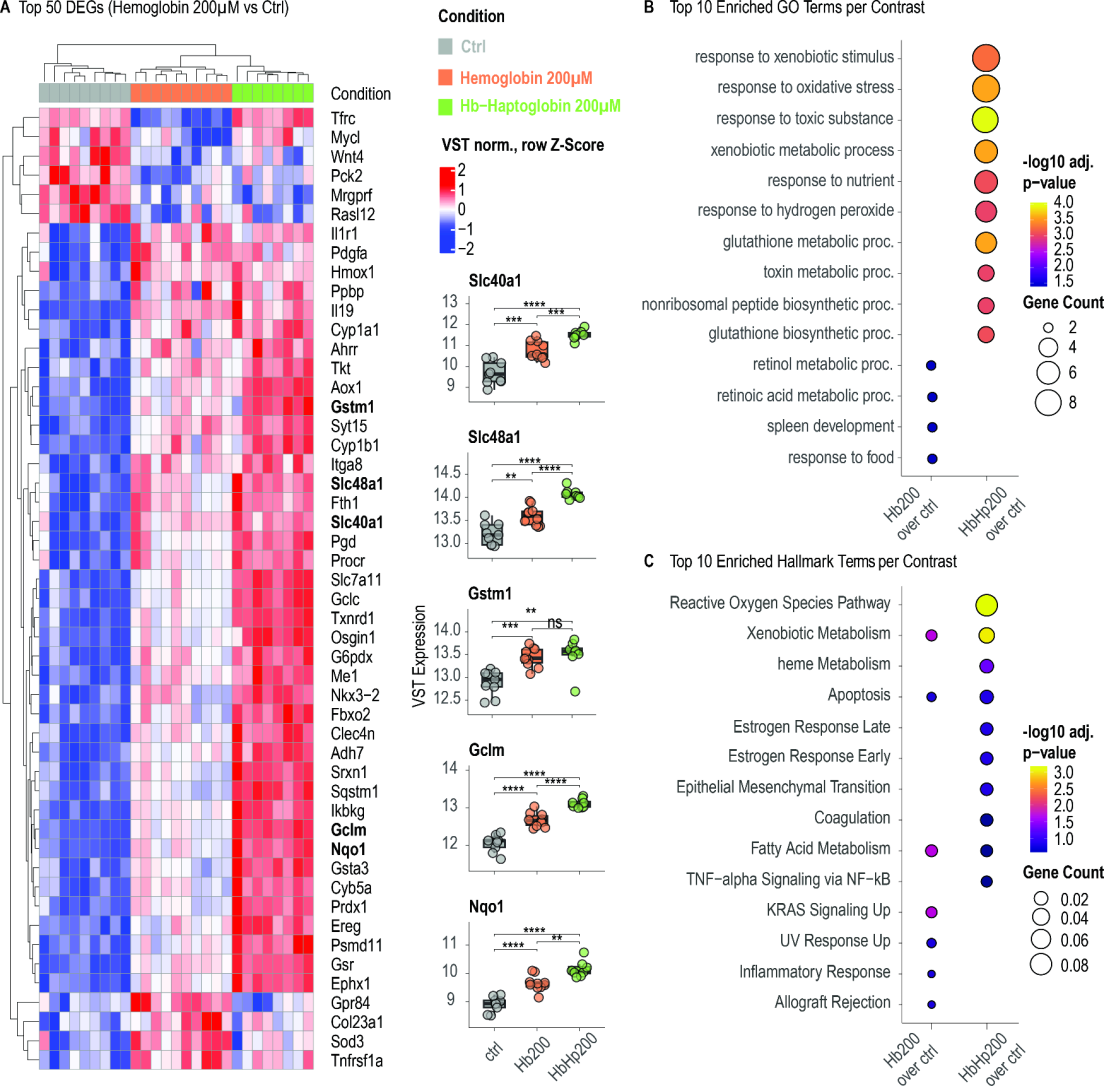
Haptoglobin amplifies oxidative stress transcriptional programs while suppressing neuroinflammatory signaling induced by cell-free hemoglobin. (A) Heatmap showing the top 50 DEGs following 48-h exposure to 200 μM Hb, HbHp complexes, or vehicle control (contrast: Hb vs control). Both conditions induced canonical oxidative stress response genes (*Nqo1*, *Gclm*, *Gstm1*) and heme–iron transporters (*Slc48a1*, *Slc40a1*), with a more pronounced response in the HbHp condition. (B) GO term enrichment analysis based on DEGs. HbHp elicited a pronounced transcriptional response, with strong enrichment across oxidative stress-related pathways. (C) Hallmark gene set enrichment analysis confirmed activation of oxidative stress and lipid peroxidation programs in both conditions.

**Figure 6:**
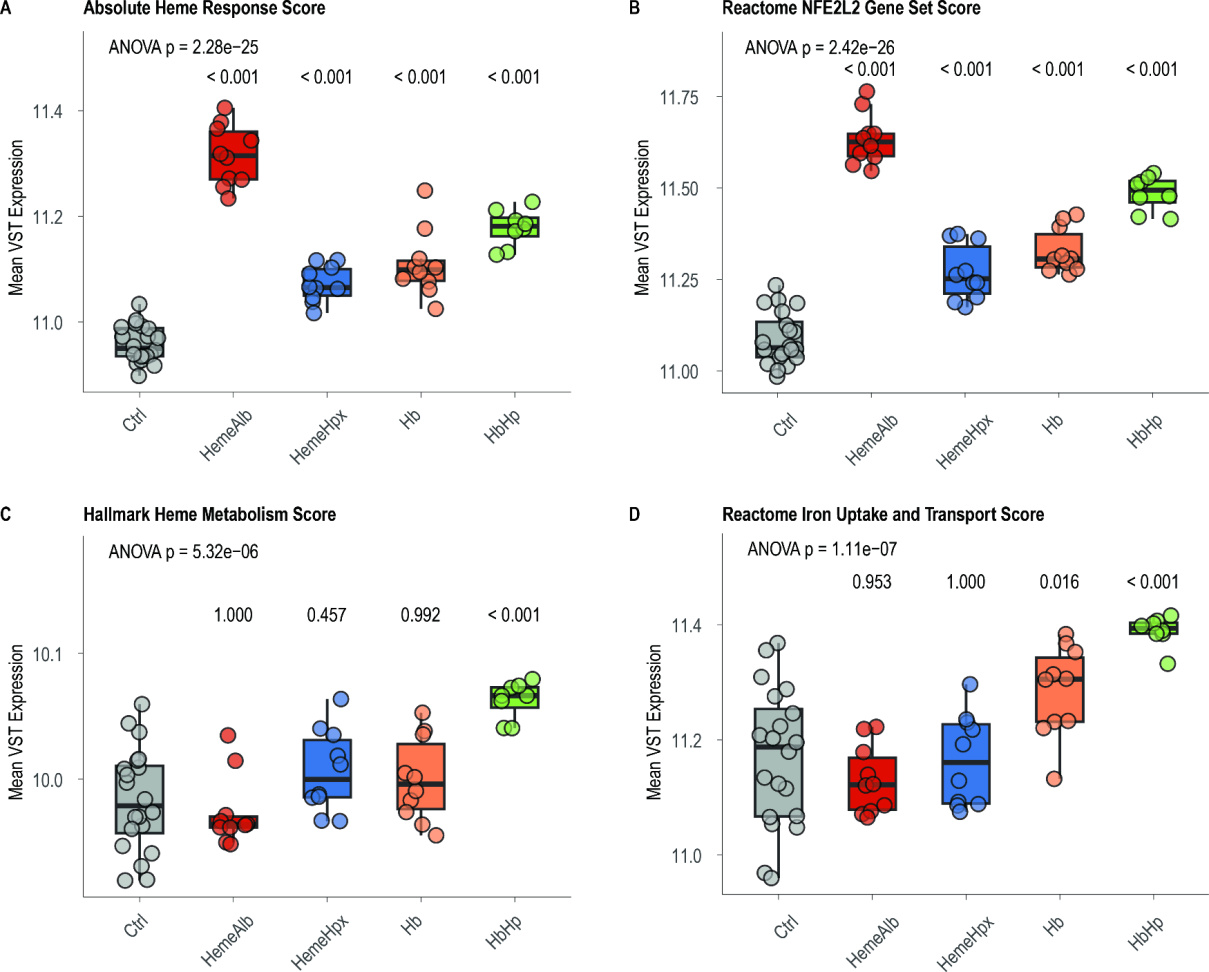
Gene-set scores separate toxic from adaptive transcriptional states. Box plots (median, IQR, individual slices) display variance-stabilized expression averages for four curated gene-set scores across conditions (*n* = 8–19). (A) Absolute heme-stress score, (B) reactome NFE2L2/NRF2 target score, (C) Hallmark heme-metabolism score, and (D) reactome iron-uptake/transport score. One-way ANOVA *p*-values are indicated per panel; pairwise Tukey *p*-values versus control are printed above each group. Free heme-albumin and Hb elevate the heme-stress score, whereas HbHp further boosts NRF2, heme-metabolism, and iron-transport scores, producing an adaptive transcriptional response enhancing cellular resilience. Heme-Hpx remains transcriptionally quiescent, consistent with the chemical sequestration of heme.

#### Responses to Heme-Albumin and HemeHpx

After 24 h of exposure, heme-albumin induced a neuroinflammatory oxidative stress signature consistent with a lipid peroxidation-triggered insult. Among the top 50 differentially expressed genes (DGEs), key transcriptome changes are reflected by induced expression of oxidative stress response (e.g., *Nqo1*, *Gclm*, *Gstm1*) and heme-iron transport (*Slc48a1*, *Slc40a1*) genes (Figure 4A). Hpx neutralized this response, redirecting enrichment to neuroendocrine Gene Ontology (GO) terms and Hallmark pathways (Figure 4B,C).

#### Responses to Hb and HbHp

Compared to heme-albumin, Hb and HbHp induced temporally delayed transcriptional responses, which were qualitatively identical but more pronounced at 48 h than at 24 h after compound exposure (Figure S1). Therefore, we utilized the 48-h exposure transcriptome for the qualitative characterization presented in Figure 5, while the 24-h data were incorporated into the cross-condition synthesis (Figure 6) and the multiparametric bootstrap analysis outlined in Figure 7. Like heme-albumin, free Hb up-regulated canonical oxidative stress response (e.g., *Nqo1*, *Gclm*, *Gstm1*) and heme-iron transport (*Slc48a1*, *Slc40a1*) genes (Figure 5A), and enriched redox-related GO and Hallmark terms (Figure 5A–C). The microglia activation markers *Gpr84* and *Tnfrsf1a* rose in parallel, consistent with a neuroinflammatory component. In contrast, HbHp further enhanced the expression of oxidative stress and heme-iron transport genes, as well as related GO and Hallmark gene sets, while suppressing *Gpr84* and *Tnfrsf1a*. This was in striking contrast to the above-described effect of Hpx, which, instead of superinducing, abandoned the heme-induced gene expression.

**Figure 7:**
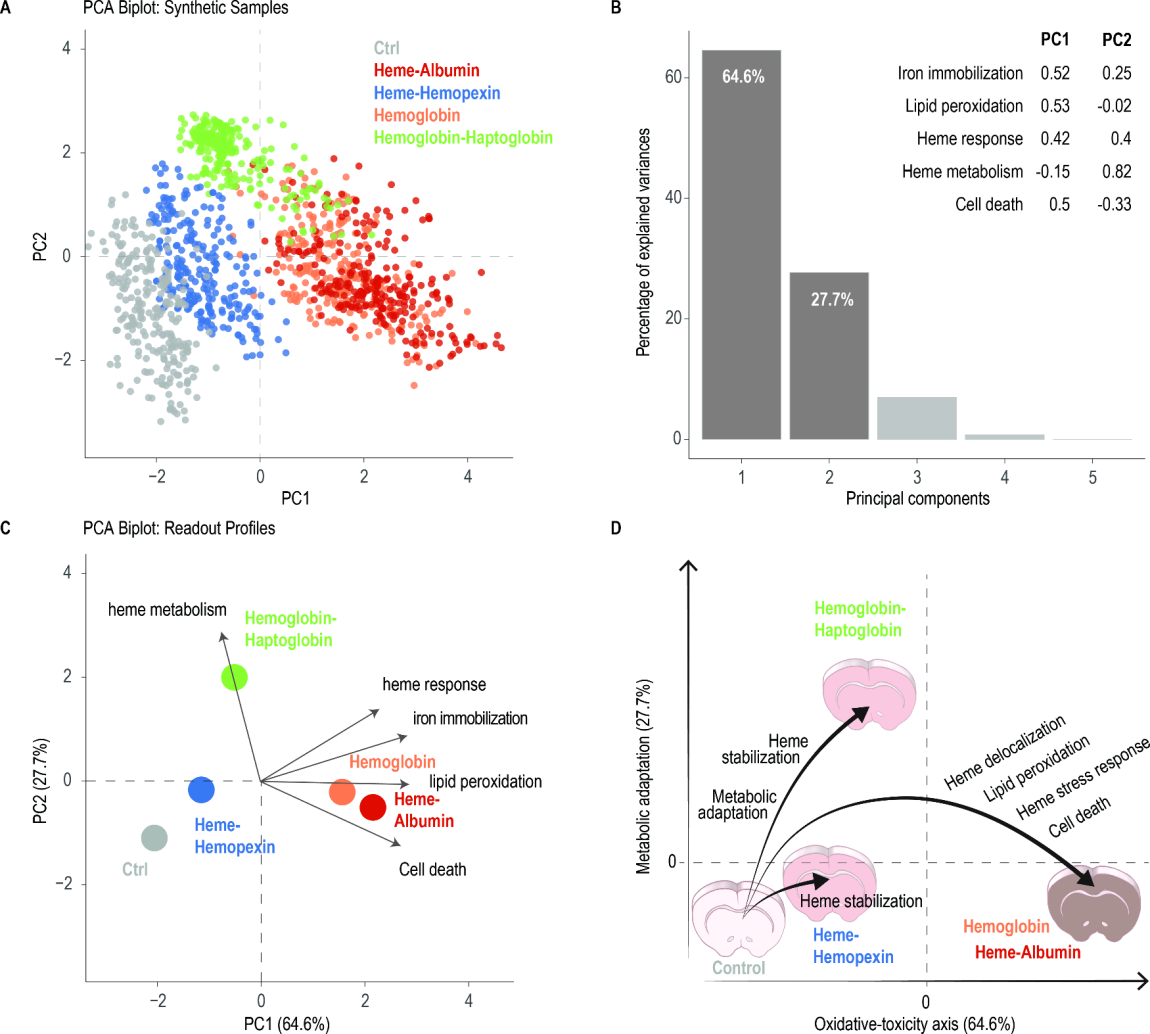
Bootstrap-PCA integrates five destructive read-outs into an oxidative-toxicity versus metabolic-adaptation landscape. (A) Scatter plot of 250 boot-strapped pseudoslices per treatment projected onto PC1 and PC2. (B) Scree plot of variance explained by the first five PCs (bars) with a loading table for the five input variables; PC1 (64.6%) is driven by ^58^Fe, TBARS, and cell death, whereas PC2 (27.7%) is dominated by the heme-metabolism score. (C) Classical biplot displaying the loadings vectors (arrows) and the centroid of each treatment after z-scaling; vectors illustrate how iron immobilization, lipid peroxidation, and cell death coload on PC1 while the adaptive transcript score loads on PC2. (D) Conceptual map translating the PCA axes into biological trajectories: uncontrolled Hb/heme chemistry pushes slices rightward along the oxidative-toxicity axis, whereas sequestration by Hpx or stabilization by Hp diverts the trajectory toward lower damage or active adaptation, respectively. The analysis links iron deposition to lipid peroxidation and neuronal death along PC1 while revealing an orthogonal Hp-triggered adaptive program on PC2.

#### Cross-Condition Synthesis

To compare transcriptional changes across all treatments, we derived a global heme-stress score from the 50 most up-regulated genes in heme-albumin-treated slices. The score rose sharply with free heme-albumin and dropped significantly when heme was neutralized by Hpx, mirroring the biochemical heme neutralization function of Hpx. Hb and HbHp yielded higher scores, with HbHp exceeding Hb, indicating enhanced transcriptional activation (Figure 6A). The same effect ranking emerged for the predefined NFE2L2/NRF2 score (Figure 6B), with a significant correlation between the two scores (*r* ^2^ = 0.97, *p* < 0.001, Figure S2A).

To disentangle the injurious from the adaptive components, we overlaid the heme-metabolism scores (Figure 6C) and iron-transport scores (Figure 6D), which again showed a significant correlation with each other (*r* ^2^ = 0.76, *p* < 0.001; Figure S2B). Free heme-albumin and Hb elevated the heme stress score without engaging metabolism. In contrast, HbHp further amplified all four scores while minimizing biochemical injury, representing protective metabolic reprogramming. This pattern suggests that intact HbHp complexes reach the cytosol and trigger an Nrf2-driven detoxification program, in contrast to the membrane-damage signaling that is induced by free heme-albumin and suppressed by Hpx.

Hence, two numerical read-outs summarize the transcriptome: heme-stress (unshielded oxidative burden) and heme-metabolism/NRF2 (adaptive detoxification). Together with iron deposition, TBARS, and cell death, they form the five-variable matrix for the bootstrap-PCA in the next section.

### Bootstrap-PCA Integrates Destructive Read-Outs into a Unified Toxicity-Adaptation Map

Because each biochemical, histological, and molecular assay consumes an individual brain slice, no single slice could be phenotyped across all five read-outs simultaneously. We therefore generated 250 bootstrapped pseudoslices per treatment by randomly sampling from the empirical distributions of (i) ^58^Fe immobilization, (ii) MDA, (iii) RNA heme-stress score, (iv) RNA heme-metabolism score, and (v) neuronal cell death index. These synthetic replicates preserve the variance structure of the source data while enabling multivariate analysis.

Principal-component analysis on the bootstrapped matrix (Figure 7A) distilled the data into two biologically coherent axes. In PC1–termed Oxidative-toxicity (64.6% variance), the highest loadings came from lipid peroxidation (0.527), ^58^Fe immobilization (0.517), and cell death (0.503), followed by the heme-stress transcript score (0.423). This axis, therefore, quantifies the sequential damage cascade originating from uncontrolled heme Fenton chemistry. PC2–termed Metabolic-adaptation (27.7% variance) was driven by the heme-metabolism score (0.819). Cell death was negatively loaded (−0.33), indicating that higher adaptive signaling is associated with improved survival.

The PCA biplot revealed four discrete attractor states: (i) Toxic – free Hb and heme-albumin, characterized by maximal iron-lipid deposition, peroxidation, and cell death. (ii) Sequestered – heme-Hpx samples centered near the control, confirming that irreversible heme sequestration largely eliminates both damage and adaptive signaling. (iii) Adaptive – HbHp displaced upward along PC2. Although residual iron loading persisted, the strong Nrf2-centered program shifted slices upward on the adaptation axis and coincided with low apoptosis. (iv) Baseline – control slices clustered at low PC1/PC2.

These results quantitatively link iron deposition to lipid peroxidation and cell death along PC1 while revealing an orthogonal HbHp-triggered adaptive detour on PC2. This provides a mechanistic map that informs our model of Hb toxicity and dual scavenger function, as discussed below.

Our study reconstructs the pathological cascade that follows erythrocyte lysis in intracerebral hemorrhage—encompassing hemoglobin oxidation, heme release, iron-driven lipid peroxidation, and neuronal death—within a single ex vivo model. By preserving neuronal–glial architecture while allowing precise toxin control, this organotypic slice approach enables direct evaluation of how scavenger proteins modulate RBC-toxin pathology.

By integrating five destructive readouts through a bootstrap-based principal component analysis (PCA), we identified two orthogonal axes. PC1 captures oxidative toxicity (iron-to-lipid ROS to cell death), while PC2 reflects a metabolic-adaptation response (dominated by Nrf2-centered transcription). Free hemoglobin and free heme-albumin cluster at the high-toxicity end of PC1, whereas their scavenger complexes segregate protectively. Heme–Hpx remains near neutral, simply neutralizing heme by tight coordination, rendering it biochemically inert, while HbHp drives slices upward on PC2, revealing a cytoprotective reprogramming that suppresses neuronal loss below baseline.

This mechanism highlights the distinction between sequential blockade and adaptive rerouting. Hpx’s quiescent heme neutralization stems from its femtomolar affinity for free heme, which prevents membrane insertion and ROS initiation. Hp, on the other hand, stabilizes Hb to reduce heme release, partially blunting PC1 and simultaneously triggering an Nrf2-dominated transcriptional program.^11,26^ This program coordinates the upregulation of iron-handling and glutathione synthesis. Overall, this response aligns with the transcriptional program of erythrophagocytic macrophages, which are the archetypal cells responsible for the safe disposal of heme and iron.^12,27^

In vivo preclinical hemorrhagic stroke models have implicated Hpx^5,28–31^ or Hp^19,32–34^ as neuroprotective, yet the cellular basis linking toxin sequestration to neuronal survival remains unproven. Genetic studies confirm Hpx’s protective role. Hpx knockouts suffer worse^19,32–34^ hemorrhagic outcomes,^29^ while Hpx overexpression shrinks hematoma and improves recovery without robustly elevating HO-1.^28^ We replicate that chemical heme-inactivation paradigm ex vivo. Hpx physically removes the heme–iron trigger and strongly suppresses ROS with minimal transcriptional engagement.

Haptoglobin, in contrast, has a dual role. It stabilizes hemoglobin dimers, limiting heme release and partially suppressing PC1. At the same time, it activates an Nrf2-dominated transcriptional program that boosts iron metabolism and enhances glutathione synthesis. As a result, slices shift upward along PC2, and neuronal cell death drops below baseline. This protective reprogramming aligns with in vivo data showing that Hp deficiency worsens hemorrhage,^26^ whereas Hp supplementation preserves vascular and neuronal integrity both in vitro and in vivo.^19,26,35^

By deploying an organotypic slice preparation that preserves neuronal–glial architecture while allowing rigorous toxin control, we now provide direct evidence that heme–iron is the quantitative driver of lipid peroxidation in the brain and that scavenger proteins either biochemically neutralize the trigger (Hpx) or convert it into an adaptive signal enhancing cellular resilience (Hp). The PCA framework formalizes these relationships and offers an intuitive map. Treatments that minimize PC1 (oxidative toxicity) while maximizing PC2 (metabolic adaptation) should yield the greatest neuroprotection.

Our observations motivate future studies to delineate how HbHp enters parenchymal cells in brain slices, traffics to intracellular compartments, and elicits the observed transcriptional and antiapoptotic effects. Consistent with bulk-phase/pinocytic uptake of plasma proteins reported in post-traumatic human brains,^36^ our findings are compatible with CD163-independent entry routes for Hb–Hp in BSCs, which lack peripheral CD163-expressing monocytes/macrophages and exhibit weak murine CD163–Hb–Hp affinity relative to the human receptor.^16,37^ Such noncanonical uptake may suffice for Hb–Hp-triggered Nrf2 responses despite limited canonical receptor availability.

Hemopexin (Hpx) sequesters free heme with subpicomolar to femtomolar affinity and is cleared via CD91/LRP1 in the liver.^21,38^ In neurons, prior reports are mixed: heme–Hpx can induce Hmox1/Nrf2-dependent programs in primary mouse cortical neurons,^21^ whereas other studies found minimal neuronal uptake and signaling.^31^ In our slice system, heme–Hpx yielded near-baseline tissue ^58^Fe accumulation and a largely quiescent transcriptome compared with hemin-albumin, consistent with chemical sequestration as the dominant effect. However, we caution against overgeneralization: (i) uptake capacity and receptor expression in BSCs differ from in vivo conditions; (ii) endocytic delivery of heme–Hpx via CD91/LRP1 may be rate-limiting in this preparation; (iii) species- and glycosylation-dependent ligand–receptor interactions can modulate signaling. Together, these factors could explain divergent heme-Hpx-driven results across studies despite clear protection at the biochemical and histological levels. A key limitation of our model is the progressive attrition of microglia in adult organotypic cultures, underrepresenting phagocytes that may contribute to hemoglobin/heme clearance in vivo.^39,40^ Another methodological trade-off refers to the selection of human proteins for our studies. Species-specific receptor–ligand interactions may affect key readouts. However, only human proteins are available at clinical-grade purity and formulation.^12,41^ In our assessment, this advantage outweighed the potential benefit of species congruence at the cost of poorly characterized and potentially contaminated mouse proteins. Within these constraints, our data support a model in which Hpx primarily neutralizes heme and minimizes PC1 (oxidative toxicity), whereas Hb–Hp stabilizes Hb and engages an NRF2-centered program that increases PC2 (metabolic adaptation)—a principle that can guide therapeutic strategies in hemorrhagic stroke.

In conclusion, we unify biochemical, transcriptomic, and histological outcomes into a coherent redox landscape that illustrates how scavenger proteins pivot RBC-toxin pathology from oxidative damage to transcriptional resilience. Hemopexin neutralizes heme chemistry, while haptoglobin orchestrates endogenous protective reprogramming. Targeting both arms thus emerges as a rational, mechanism-based strategy to mitigate neuronal injury in intracerebral hemorrhage.

## Materials and Methods

### Animals

Wild-type C57BL/6J mice (male and female, 6–8 weeks) and CAG-Luc mice (B6;FVB-Ptprca Tg­(CAG-luc,-GFP)­L2G85Chco Thy1a/J, JAX #025854) were bred and housed in the University of Zurich Laboratory Animal Service Center (LASC). All procedures were approved by the Swiss Federal Veterinary Office (license ZH96/2023).

### Organotypic Brain-Slice Culture

Coronal BSCs were prepared essentially as described by Stoppini et al. (1991) with minor adaptations.^42–46^ Briefly, deeply anesthetized mice were transcardially perfused with ice-cold phosphate-buffered saline (PBS, Gibco 17502048). Brains were removed, glued to the vibratome stage, and sectioned coronally (150 μm; speed 4, frequency 10) into Hibernate-A at 4 °C (<15 min). Two slices were placed on each PTFE insert (0.4 μm, Millicell) in 6-well plates containing 1.1 mL plating medium (Neurobasal-A + 20% heat-inactivated horse serum + antibiotic-antimycotic). Cultures were maintained at 37 °C, 5% CO_2_. On day 4, the medium was replaced with serum-free growth medium (Neurobasal-A supplemented with N-2, B-27 Plus, G-5, and antibiotic-antimycotics). Experiments were performed on day 7 or, for longer cultivation, after a half-medium change.

### Preparation of RBC Toxins and Scavenger Complexes

All hemoglobin, heme and their scavenger proteins (hemopexin, haptoglobin) used in this study were of human origin, except for isotopic labeled hemoglobin and heme that were murine.

#### Heme-Albumin

Hemin (Frontier Scientific) was dissolved in 100 mM NaOH, mixed with 20% human albumin (CSL Behring, lot P100634753) to 4 mM, pH-adjusted to 7.45 with 144 mM H_3_PO_4_, brought to 25 mL with H_2_O and sterile-filtered; stable at 4 °C for ≤ 1 week.

#### Heme-Hemopexin

Hemin (24 mg in 2.63 mL 100 mM NaOH) was mixed at a 1:1.1 molar ratio with hemopexin (92 mg/mL; CSL Behring, lot T0342022B) to ensure complete binding, sterile-filtered, and used within 24 h. We used clinical-grade human Hp, Hpx and albumin for all functional exposures to ensure consistent protein quality and formulation suitable for translational work. We acknowledge that species and glycosylation differences may influence uptake and signaling.

#### Hemoglobin

Stroma-free human Hb was purified from expired blood units as described.^14^ Hb was complexed with human plasma-derived Hp 1-1 (CSL Behring, specifications on request) at a 1:1.1 molar ratio; complex formation was verified by HPLC size exclusion chromatography (Agilent).

### Isotopically Labeled Heme and Hemoglobin

Ferric carboxymaltose enriched with ^58^Fe was synthesized as described,^24^ washed, and resuspended (1.5 mg/mL NaCl 0.9%). Eight-week C57BL/6J mice were fed an iron-deficient diet (<0.5 mg Fe/kg) for 3 weeks, then injected i.v. with 150 μg ^58^Fe-FCM five times over 2 weeks. After a 2-week incorporation period, blood was collected and ^58^Fe-Hb purified. Heme was isolated by acetone precipitation and ethyl acetate extraction, as described by Egyed.^47^

### Viability Assays

#### Bioluminescence

BSCs from CAG-Luc mice were incubated with 25 μM DMNPE-caged luciferin (GoldBio L13010) for 30 min; Bioluminescent signal intensity was measured for 30 s on a ChemiDoc system (Bio-Rad, Image Lab 5.2.1).

#### Lactate Dehydrogenase (LDH) Release

LDH was quantified in the culture medium using the CyQUANT LDH kit (Invitrogen C203000), read at 560/590 nm (Tecan Infinite M200 Pro).

#### Glucose Oxidase (GOx) Challenge

Oxidative stress was imposed with 6.25–100 mUmL^–1^ glucose oxidase added directly to the culture medium (Sigma, G7141–10KU).

### ^58^Fe Quantification by ICP-MS

Slices were digested in 200 μL 67% HNO_3_ (1 h incubation at room temperature, then 1 h sonication at 80 °C) by subsequent addition of 50 μL of 30% H_2_O_2_ (1 h incubation at room temperature, then 1 h sonication at 80 °C). Digests were diluted to 4% HNO_3_ and analyzed using an Agilent 8800 triple-quadrupole inductively coupled plasma mass spectrometry (ICP-MS) instrument (Department of Chemistry, University of Zurich).

### Lipid Peroxidation (TBARS)

Two slices per insert were pooled, homogenized in 150 μL of RIPA (Thermo 89900), and assayed for thiobarbituric acid-reactive substances (TBARS) as described.^48^ In brief, 100 μL homogenate was mixed with 500 μL 750 mM trichloroacetic acid in 1 M HCl, followed by 400 μL 25 mM 2-thiobarbituric acid in 1 M NaOH. Tubes were sealed and incubated for 60 min at 80 °C, cooled on ice for 5 min, and centrifuged at 15,000*g* for 5 min. Absorbance measured at 532 nm with background subtraction at 600 nm (Infinite M200 Pro, Tecan). TBARS concentration (μM malondialdehyde equivalents) was calculated using ε = 156 mM^–1^ cm^–1^ and normalized to total protein determined by BCA (Thermo Pierce).

### Histology and Image Analysis

Slices were fixed in 4% PFA (overnight, 4 °C) and permeabilized in permeabilization buffer (PB, 1% BSA/0.5% Triton X-100; 12 h, 4 °C) before immunostaining. Primary antibodies (72 h, 4 °C, in PB: PBS 1:1): guinea-pig anti-NeuN 1:1000 (Synaptic Systems 266 004), rabbit anticleaved Caspase-3 1:300 (Cell Signaling Technology #9661), rat anti-GFAP 1:300 (Thermo Fisher 13-0300). Secondary antibodies (24 h, 4 °C, in PB:PBS 1:1): goat antiguinea-pig Alexa 488 1:300 (Invitrogen A-11073), goat antirabbit Alexa 555 1:300 (Invitrogen A-21429), goat antirat Alexa 647 1:300 (Invitrogen A-21247). Slices were counterstained with Hoechst 33342 1:2000 (Invitrogen H3570) and mounted in ProLong Gold Antifade (Thermo Fisher P36930).

Images were acquired on a Leica TCS SP8 inverted confocal microscope equipped with a 20×/0.75 NA HC PL APO objective (Center for Microscopy and Image Analysis, University of Zurich). Tile scans (single optical section at slice mid-depth) were exported as 16-bit TIFFs.

Image visualization and preprocessing were performed using Fiji;^49^ single-cell segmentation and fluorescence quantification were accomplished with a custom Python pipeline (Python 3.12.10). Cells were clustered by *k*-means (*k* = 2) on log-transformed NeuN and Caspase-3 intensities to classify Casp3^high^/NeuN^low^ (apoptotic) and Casp3^low^/NeuN^high^ (viable) populations. Downstream statistics and plotting were performed in R 4.4.2 (2024-10-31). AI-based tools were used to improve code efficiency in Python and R. All analyses and interpretations were carried out by the authors.

### Bulk RNA-seq and Gene-Set Scoring

RNA was extracted (Lexogen SPLIT kit), quality-checked (Agilent TapeStation), and sequenced (Illumina NovaSeq X Plus/6000; TruSeq mRNA) at the Functional Genomics Center Zurich (FGCZ) of the University of Zurich and ETH Zurich. Differential expression analysis was performed using DESeq2 ^50^ with LFC apeglm shrinkage method.^51^ To quantify the transcriptional response to RBC toxins and scavenger proteins, we calculated a “heme response score” derived from the top 50 upregulated DEGs in BSC treated with 200 μM heme-albumin compared to medium-only control.

### Principal-Component Analysis

For each condition (Ctrl, heme–albumin, heme + Hpx, Hb, Hb + Hp), we generated 250 bootstrap replicates by sampling with replacement from the empirical distributions of the five readouts (^58^Fe immobilization, MDA, heme‑stress score, heme‑metabolism score, neuronal cell‑death index). Each bootstrap replicate comprised one draw per readout. The resulting 1,250 × 5 matrix (five conditions × 250 replicates) was Z‑scaled prior to PCA (prcomp­()). The proportions of explained variance were examined using a scree plot, focusing on the first two principal components (PC1 and PC2), which accounted for the majority of the multivariate variation. AI-based tools were used to improve code efficiency. All analyses and interpretations were performed by the authors.

### Statistics

All statistical analyses were performed in R 4.4.2 (2024-10-31) using functions from the tidyverse package.^52^ Data visualization was performed using ggplot2.^53^ *P*-values <0.05 were considered statistically significant. AI-based tools were used to refine code syntax. Data analysis and statistical interpretation were done by the authors.

## Supplementary Material



## Data Availability

The sequencing data sets generated and analyzed during the current study are available in the Gene Expression Omnibus repository (GSE299269). Supporting Information, including the original data, a Python notebook for the pseudocytometric analysis, and the R code to reproduce all analyses and figures in this publication, are available online (10.5281/zenodo.15530642). To review GEO accession GSE299269: Go to https://www.ncbi.nlm.nih.gov/geo/query/acc.cgi?acc=GSE299269.

## Notes

Transcriptional response after 24 and 48 h of Hb exposure. Correlation of gene set scores (PDF)
