# Supplementary material for: Haptoglobin and Hemopexin Redirect Heme-Driven Oxidative Stress and Neurotoxicity in Organotypic Brain Slices
Source: ACS Chem Neurosci. 2025 Dec 19;17(1):77–89. doi: 10.1021/acschemneuro.5c00511 (PMC12784396; doi:10.1021/acschemneuro.5c00511)
Supplement: Supplementary file 1 [file cn5c00511_si_001.pdf]

Supplementary Figures

Haptoglobin and Hemopexin Redirect Heme-Driven Oxidative Stress and Neurotoxicity in Organotypic Brain Slices

Running title: Hp and Hpx redirect heme toxicity in the brain

Anna-Lea T. Stalder[a], Raphael M. Buzzi[a], Dominik J. Schaer[a]

[a] Department of Internal Medicine, Universitätsspital and University of Zurich; Zurich, Switzerland

Supplementary Figure 1

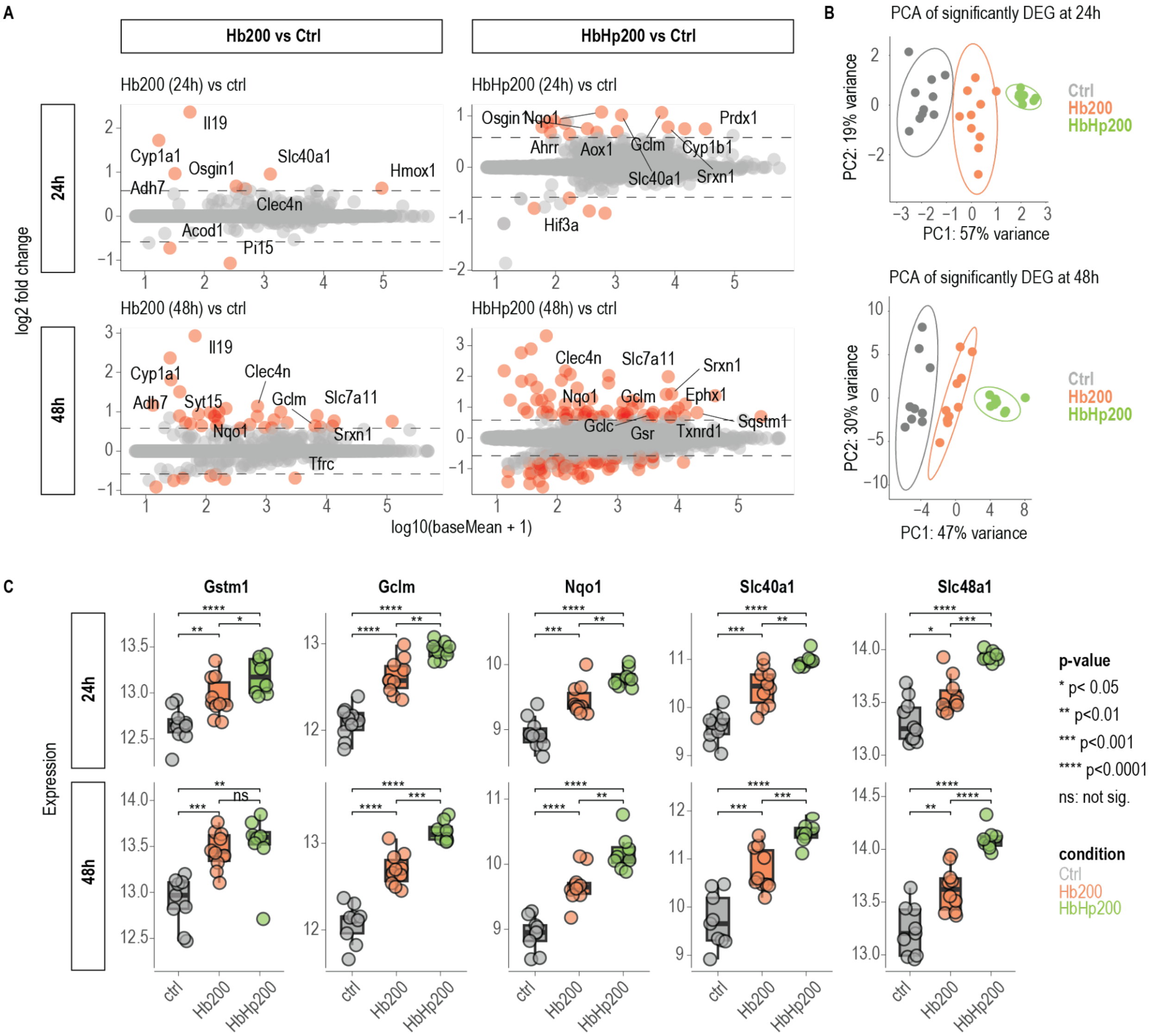

Supplementary Figure 1: Transcriptional response pattern is similar after 24 and 48 hours of Hb exposure and more pronounced at 48 hours.

(A) MA plots of Hb (200µM) vs. Ctrl and HbHp (200µM) vs. Ctrl at 24h and 48h after exposure show enhanced transcriptional response after 48h.

(B) PCA plots of significant DEG at 24h and 48h show a similar distribution pattern

(C) Oxidative stress response genes (*Nqo1*, *Gclm*, *Gstm1*) and heme–iron transporter genes (*Slc48a1*, *Slc40a1*) show similar expression patterns at 24h and 48h, with higher overall gene expression at 48h.

Supplementary Figure 2

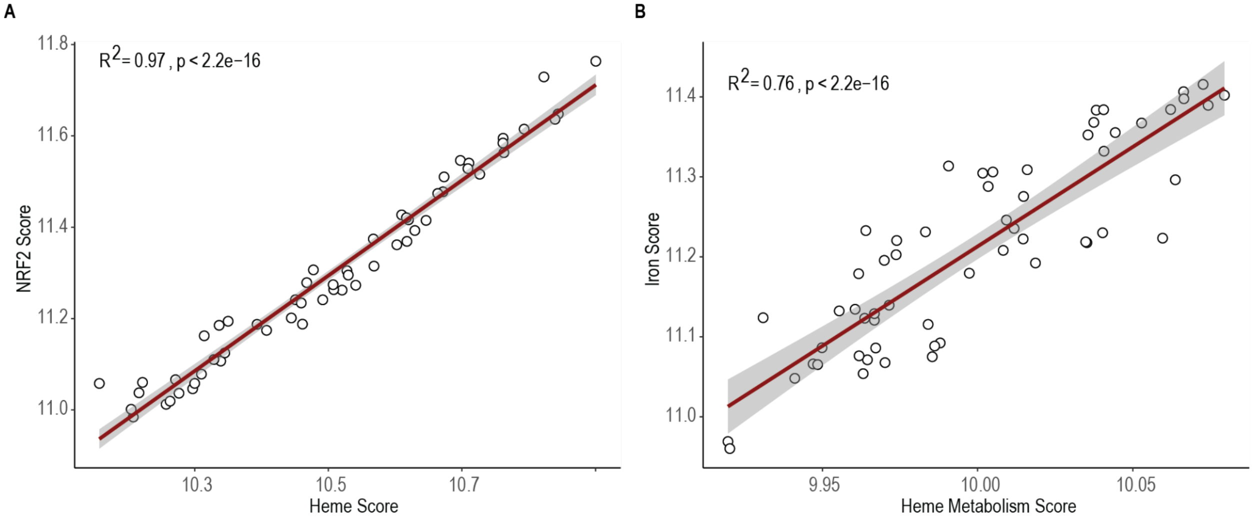

Supplementary Figure 2: Correlation of Gene Set Scores.
(A) Correlation of heme response score and Reactome Nrf2 score, r2 = 0.97, p < 0.0001
(B) Correlation of Hallmark heme metabolism score and Reactome iron uptake and transport score, r2 = 0.76, p < 0.0001
